# Herbicide Application Under Co-Cultivation Is Associated with Early Microbiome Assembly Shifts and Later Physiological Decline in Rice

**DOI:** 10.3390/microorganisms14051137

**Published:** 2026-05-17

**Authors:** Yingxi Li, Mingfeng He, Yao Song, Lu Liu, Jiling Xiao, Jie Wang, Bin Yang, Shunyi Ouyang, Xin Li, Di Peng, Zheyuan Zhu

**Affiliations:** 1 Longping Agricultural College, Hunan University, Changsha 410017, China; liyingxi@hnu.edu.cn; 2 Hunan Institute of Microbiology, Hunan Academy of Agricultural Sciences, Changsha 410125, China; liulu@hunaas.cn (L.L.); jilingxiao@hunaas.cn (J.X.); jiew@hunaas.cn (J.W.); thisyangbin@hunaas.cn (B.Y.); s2007203272@yeah.net (X.L.); 3 Yuelushan Laboratory, Changsha 410001, China; 4 College of Plant Protection, Hunan Agricultural University, Changsha 410128, China; hemingfeng168@stu.hunau.edu.cn (M.H.); hnaupp_sy@stu.hunau.edu.cn (Y.S.); ouyang777@stu.hunau.edu.cn (S.O.)

**Keywords:** rhizosphere microbiome, microbial community assembly, herbicide selectivity, propanil, cyhalofop-butyl

## Abstract

Herbicides considered selective to rice are generally evaluated based on their direct crop safety and weed suppression effects, yet it remains unclear whether they may also trigger indirect or context-dependent effects on rice under rice–barnyardgrass co-cultivation. To address this question, we compared rice performance and associated microbial dynamics under six conditions: rice–barnyardgrass co-cultivation and rice monoculture, each treated with a water spray control or sublethal doses of propanil (Pro, 66.7 mg a.i. L^−1^) or cyhalofop-butyl (Cyh, 5.86 mg a.i. L^−1^). Barnyardgrass exhibited visible injury and stronger leaf-level oxidative stress responses, whereas rice displayed no discernible phytotoxic symptoms. Nevertheless, under co-cultivation, herbicide treatment significantly suppressed rice growth, with up to 17.8% lower root lengths and 24.8% lower shoot fresh weights, with reductions varying by herbicide and trait. By contrast, comparable suppression was not observed under herbicide exposure or co-cultivation alone, identifying this response as an emergent, context-dependent negative effect. Microbiota reassembly emerged as an early and stage-specific component of the herbicide-associated response under co-cultivation, with the most pronounced changes detected on day 5 and occurring primarily in bacterial communities. Moreover, bacterial community variation was negatively correlated with root length (ρ = −0.664), and urease activity declined under herbicide treatment. Together, these findings indicate that in paddy fields, herbicides act not only on individual plants but also as an external disturbance to the coupled rice–barnyardgrass system, for which microbiota reorganization represents a key component of the ecological response. Our results suggest that herbicide selectivity should be interpreted within a crop–weed–microbiome context, rather than being inferred solely from their direct crop safety and weed suppression effects.

## 1. Introduction

Rice production is strongly constrained by barnyardgrass (*Echinochloa crus-galli*), one of the most destructive weeds in paddy fields worldwide because of its rapid growth, strong adaptability, and substantial niche overlap with rice [1,2]. Because chemical control remains the principal strategy for barnyardgrass management, herbicides considered selective to rice are widely used in paddy systems. However, their selectivity is typically evaluated through their direct crop safety and weed suppression effects, and it remains unclear whether they may also trigger indirect or context-dependent effects on rice under rice–barnyardgrass coexistence. Addressing this problem is important because competition between rice and barnyardgrass begins early in development and may extend beyond direct resource capture to broader ecological interactions that affect crop performance [3].

Crop–weed competition has traditionally been viewed as competition for light, water, and mineral nutrients [4,5]. Increasing evidence, however, indicates that plant-associated microbiota also influence plant stress responses and competitive outcomes, particularly in the rhizosphere and root interior [6]. Plant-associated microbial communities respond dynamically to plant status and environmental perturbation [7], while root exudation and host filtering further shape their assembly [8,9]. Accordingly, crop–weed coexistence can be viewed as a coupled belowground system in which soil biota, microbial assembly, and plant–soil feedback jointly influence ecological interactions and competitive outcomes [10,11].

This systems perspective is especially relevant in paddy fields, where rice and barnyardgrass coexist in close proximity throughout early growth. Previous studies have shown that rice can respond to barnyardgrass stress by recruiting specific rhizosphere or endophytic taxa, such as *Sphingomonas* and *Azospirillum* [12,13]. Barnyardgrass, in turn, can exhibit inducible allelopathic responses during mixed culture with rice [14]. Evidence from related paddy competition systems further suggests that such interference may be accompanied by shifts in microbial composition, interaction patterns, and rhizosphere ecological functions [15]. However, most studies on rice–barnyardgrass interactions have focused on allelochemicals, plant metabolic responses, or static community descriptions, whereas much less is known about how external disturbances reshape this crop–weed system through microbial assembly, microbial associations, and rhizosphere biochemical responses [14,16].

This gap is significant because paddy fields are not only biological interaction systems but also intensively managed agroecosystems. Among the most common anthropogenic disturbances in these systems is herbicide application, which is widely used in rice production to control barnyardgrass [17]. Propanil and cyhalofop-butyl are widely used paddy herbicides that are effective against barnyardgrass and are generally regarded as selective and safe for rice at recommended doses [18]. We selected these two herbicides because they are agronomically relevant for barnyardgrass control and differ in their action mechanisms, with propanil acting mainly as a contact herbicide and cyhalofop-butyl as a systemic herbicide. Their apparent crop safety is partly attributed to the detoxification capacity of rice: propanil selectivity is associated with rapid hydrolysis by aryl acylamidase, whereas cyhalofop-butyl selectivity is related to its breakdown into less toxic or inactive forms in rice [19,20]. Their agronomic selectivity is commonly reflected in their effectiveness in weed control and in limited visible crop injuries.

Studies on rice monoculture or simplified soil systems often suggest that herbicide-induced disturbances to rice performance or soil biological properties are limited, dose-dependent, or transient [21]. Although herbicides can alter microbial groups or functional diversity in paddy soils, these microbial responses are rarely examined within active coexisting crop–weed systems. Moreover, assessments of propanil and cyhalofop-butyl at recommended doses have largely focused on crop phenotype, weed mortality, or single-soil responses, with limited consideration of the coupled interactions that arise when rice and barnyardgrass coexist [22,23,24,25]. Under co-cultivation, herbicides may instead disturb the belowground system jointly shaped by rice, barnyardgrass, and their associated microbiota.

Such a perturbation may produce effects beyond weed suppression, including changes in microbiota assembly, microbial associations, and rhizosphere function-related responses. Addressing this possibility requires going beyond single time-point community descriptions to examine temporal changes in microbial diversity, assembly processes, cross-kingdom associations, and soil enzyme activities [26]. Based on this rationale, we established rice monoculture and rice–barnyardgrass co-cultivation systems and applied sublethal levels of propanil and cyhalofop-butyl. Unlike previous studies, which mainly evaluated herbicide selectivity in rice monoculture, simplified soil systems, or field efficacy contexts, this study considered herbicide exposure as a disturbance within an active rice–barnyardgrass–microbiome system. The study asks whether, beyond weed suppression and apparent crop safety, herbicide application perturbs the rice–barnyardgrass co-cultivation system and elicits broader responses in rice and its associated belowground environment.

## 2. Materials and Methods

### 2.1. Experimental Design and Sampling

The pot experiment was conducted outdoors in Changsha, Hunan Province, China (28°11′23″ N, 113°04′20″ E). The pots were maintained under ambient light, temperature, and photoperiod conditions, with water levels manually controlled throughout the experiment. Both rice–barnyardgrass co-cultivation and rice monoculture were treated with water spray control (CK), propanil (Pro), or cyhalofop-butyl (Cyh), resulting in six treatment conditions. Pro and Cyh were selected as barnyardgrass-control herbicides with contrasting behaviors: Pro is mainly contact-acting, whereas Cyh is systemic. Propanil was applied as a water-dispersible granule formulation (80% active ingredient; Syngenta Crop Protection Co., Ltd., Shanghai, China), and cyhalofop-butyl as an emulsifiable concentrate (100 g a.i. L^−1^; Corteva Agriscience, Shanghai, China). Preliminary concentration-screening assays were conducted to determine suitable sublethal treatment levels. Working solutions were 66.7 mg a.i. L^−1^ Pro and 5.86 mg a.i. L^−1^ Cyh, each causing approximately 30% fresh-weight reduction in the barnyardgrass population used. These concentrations were selected as reduced sublethal treatment levels, imposing moderate herbicide stress without severe direct injury to rice. They do not represent maximum recommended field doses. Rectangular plastic pots (300 mm × 200 mm × 87 mm) were filled with a 1:1 (*v*/*v*) mixture of field soil and commercial seedling substrate composed mainly of peat, coconut coir, perlite, and vermiculite. The field soil had not been exposed to Pro or Cyh for at least five years. The soil–substrate mixture had the following properties: pH 5.08, organic matter 69.3 g kg^−1^, and cation exchange capacity 11.2 cmol(+) kg^−1^. No additional fertilizer was applied. Barnyardgrass seeds were collected from a single field population in Henan, China, and the same seed batch was used throughout the experiment. Rice seeds of IR64 were provided by the Hunan Hybrid Rice Research Center. These seeds were surface-sterilized with 70% ethanol and 1% sodium hypochlorite, rinsed three times with sterile water, and germinated in the dark for 24 h. Uniformly germinated rice and barnyardgrass seeds were directly sown into pots according to treatment arrangement. Each treatment consisted of 16 pots in total, with eight plants per pot. These 16 pots were allocated to four destructive sampling time points, with four pots randomly harvested per treatment at each time point. Co-cultivation pots contained four rice and four barnyardgrass plants arranged alternately, whereas monoculture pots contained eight rice plants to maintain equal plant density. Pots were randomized, repositioned periodically to minimize edge effects, and irrigated with tap water to maintain moist soil without standing water. No other pesticides were applied, and non-target weeds were removed manually.

Herbicides were applied approximately 14 d after sowing in pots when rice seedlings were at the two-leaf-and-one-heart stage and barnyardgrass seedlings were at the 3–4-leaf stage. Working solutions were evenly sprayed onto foliage with a hand-held sprayer until the leaves were fully soaked without runoff; CK plants were sprayed the same volume of water. Irrigation was withheld immediately before and after application to minimize wash-off. Leaf samples were collected at 24 h after application for physiological assays. Rhizosphere soil and root samples were collected separately from rice and barnyardgrass at 1, 3, 5, and 7 d after herbicide application. Because sampling was destructive, different pots were used at each time point. At each time point, four pots per treatment were used as biological replicates (*n* = 4). For rhizosphere soil, root, and enzyme analyses, four plants from the same pot were pooled as one pot-level replicate. In co-cultivation pots, rice and barnyardgrass samples were collected and processed separately. Rhizosphere soil was collected by gently removing loosely attached soil; the soil tightly adhering to the root surface was retained. Soil samples were transported to the laboratory on dry ice and stored at −80 °C until analysis.

### 2.2. Physiological and Soil Enzyme Assays

Samples of rice and barnyardgrass leaves were collected 24 h after herbicide application to measure SOD, POD, and CAT activities and MDA content using commercial assay kits [27,28,29,30]. Because leaf biomass was limited at this seedling stage, three biological replicates were used for each treatment. Plant height, shoot fresh weight, root length, and SPAD value were measured at 1, 3, 5, and 7 d after application. Plant height, shoot fresh weight, and root length were recorded for individual plants, and SPAD values were determined using a handheld chlorophyll meter as a non-destructive indicator of relative chlorophyll status. Plant-level measurements were averaged by pot. Root architectural traits were assessed using one randomly selected plant from each biological replicate at each sampling time. Roots were gently washed of adhering soil, evenly spread in a transparent root tray, and scanned at 900 dpi using a Microtek ScanWizard EZ scanner (Hangzhou Wanshen Detection Technology Co., Ltd., Hangzhou, China). Images were analyzed using the LA-S root analysis software supplied with the LA-S root analysis system (Hangzhou Wanshen Detection Technology Co., Ltd., Hangzhou, China; version number not specified by the manufacturer; product information available at: http://wseen.com/ProductDetail.aspx?id=9&classid=29) to determine root surface area, total root volume, average root diameter, and root tip numbers in diameter classes of 0–0.2, 0.2–0.5, 0.5–1, 1–2, and >2 mm.

Urease and acid phosphatase activities were determined in rice and barnyardgrass rhizosphere soils using commercial assay kits (S-UE, D799506; S-ACP, D799518-0100) according to the manufacturer’s instructions [31,32]. Briefly, soil samples were air-dried or dried at 37 °C, sieved through a 30–50-mesh sieve, and then incubated with the corresponding reagents at 37 °C for 24 h. For urease and acid phosphatase assays, 0.05 g and approximately 0.10 g of air-dried soil were used, respectively; after incubation, samples were centrifuged at 10,000× *g* for 10 min and the supernatants were used for colorimetric measurement. Absorbance was measured in 96-well plates at 630 nm for urease and 660 nm for acid phosphatase using a microplate reader (Thermo Fisher Scientific, Waltham, MA, USA). Each batch included blanks, standards, and samples. For urease activity, ΔA_sample was calculated as A_measurement − A_control, and ΔA_standard was calculated as A_standard − A_blank. For acid phosphatase activity, ΔA_sample was calculated as A_sample − A_blank, and ΔA_standard was calculated as A_standard − A_blank. Enzyme activity was calculated as (ΔA_sample/ΔA_standard) × C_standard × D/m, where C_standard is the standard concentration, D the dilution factor, and m the soil mass. Enzyme activities were expressed as U g^−1^ air-dried soil.

### 2.3. DNA Extraction and Amplicon Sequencing

Total community DNA was extracted from rhizosphere soil and from root samples surface-sterilized for endophytic community analysis using an E.Z.N.A. MagBind Soil DNA Kit (Omega Bio-tek, Norcross, GA, USA). DNA concentration was measured using a Qubit 4.0 fluorometer (Thermo Fisher Scientific, Waltham, MA, USA). The bacterial 16S rRNA V3–V4 region was amplified using primers 341F (5′-CCTACGGGNGGCWGCAG-3′) and 806R (5′-GACTACHVGGGTATCTAATCC-3′). The fungal ITS1–ITS2 region was amplified using primers ITS1F (5′-CTTGGTCATTTAGAGGAAGTAA-3′) and ITS2R (5′-GCTGCGTTCTTCATCGATGC-3′). PCR amplification was performed using 2× Hieff Robust PCR Master Mix (Yeasen Biotechnology (Shanghai) Co., Ltd., Shanghai, China) on an Applied Biosystems 9700 thermal cycler (Applied Biosystems, Foster City, CA, USA). The PCR program was 95 °C for 3 min; 5 cycles of 95 °C for 30 s, 45 °C for 30 s, and 72 °C for 30 s; 20 cycles of 95 °C for 30 s, 55 °C for 30 s, and 72 °C for 30 s; and 72 °C for 5 min. PCR products were analyzed by 2% agarose gel electrophoresis and purified using Hieff NGS DNA Selection Beads (Yeasen Biotechnology (Shanghai) Co., Ltd., Shanghai, China). Purified amplicons were indexed, quantified using a Qubit 4.0 dsDNA assay, quality-checked using an Agilent 2100 Bioanalyzer (Agilent Technologies, Santa Clara, CA, USA), pooled at equimolar concentrations, and sequenced on an Illumina MiSeq platform (Illumina, San Diego, CA, USA). Amplicon sequencing was performed by Sangon Biotech (Shanghai) Co., Ltd. (Shanghai, China).

### 2.4. Bioinformatic Analysis of Amplicon Sequencing Data

Raw paired-end reads were merged using PEAR (v0.9.8) and quality-filtered. High-quality sequences were clustered into operational taxonomic units (OTUs) at a 97% identity threshold using the UPARSE algorithm within USEARCH (v11.0.667). Chimeras and singleton OTUs were removed, and the most abundant sequence in each OTU was used as the representative sequence. Bacterial and fungal OTUs were annotated against the SILVA and UNITE databases, respectively, and chloroplast and mitochondrial sequences were removed from the bacterial dataset. OTUs retained for diversity analyses were required to have at least 10 reads in at least two samples. Sequencing depth was evaluated using OTU rarefaction curves, which approached saturation for the retained samples (Figure S1).

Filtered OTU tables were rarefied to the minimum sequencing depth within each dataset before alpha- and beta-diversity analyses. Chao1 and Shannon indices were calculated from rarefied OTU tables. Beta diversity was estimated using Bray–Curtis dissimilarity matrices calculated from the rarefied OTU tables, and differences in community composition among treatments were tested by PERMANOVA using the adonis function in the R package vegan version 2.6-4 under R version 4.5.3 with 999 permutations. The beta nearest taxon index (βNTI) was calculated using the corresponding rarefied OTU tables to quantify phylogenetic turnover relative to null expectations. βNTI > 2 indicated variable selection, βNTI < −2 indicated homogeneous selection, and |βNTI| < 2 indicated stochastic-dominated turnover. The relative contributions of inferred assembly categories were summarized from pairwise comparisons, with RCbray used to assist the classification of stochastic categories. For visualization, drift- and dispersal-related stochastic categories were combined as DDH, and the contribution of each category was calculated as the percentage of pairwise comparisons assigned to that category.

Bacterial–fungal interkingdom networks were constructed at the genus level using Spearman correlations, retaining links with |ρ| > 0.8. Cutoff sensitivity was assessed using alternative thresholds of |ρ| > 0.7 and |ρ| > 0.9, and link stability was evaluated by leave-one-out recalculation. Network topological properties were calculated for comparison among treatments. Differentially abundant genera were identified in R using DESeq2 based on unrarefied genus-level count tables, with normalization performed internally by DESeq2. Pairwise comparisons were performed separately for rice and barnyardgrass samples under co-cultivation conditions. Shared differential genera between hosts were extracted, and their log2 fold changes were used to classify co-up, co-down, and opposite response patterns. Unless otherwise stated, data analysis and visualization were performed in R.

### 2.5. Statistical Analysis

Unless otherwise stated, non-amplicon statistical analyses were performed using GraphPad Prism 9.5. Data are presented as means ± SD, and *p* < 0.05 was considered statistically significant. For pot-based measurements, the pot was the experimental unit, and plant-level measurements within each pot were averaged before analysis. Before ANOVA, residual normality and homogeneity of variances were assessed using the Shapiro–Wilk test and Brown–Forsythe test, respectively. For time-course data, two-way ANOVA was performed with treatment and sampling time as fixed factors. Because a destructive sampling design was used and different pots were harvested at each time point, the sampling time was not treated as a repeated-measures factor. At individual time points, treatment effects were tested by one-way ANOVA followed by Dunnett’s test against the corresponding CK. To evaluate whether the effects of herbicide on rice growth depended on cultivation pattern, two-way ANOVA was performed with cultivation pattern and herbicide treatment as fixed factors, and effect size was expressed as η^2^. For the association between bacterial community variation and early rice root response, PCoA1 scores were extracted from the Bray–Curtis PCoA of day-5 co-cultivated rice rhizosphere bacterial communities. The percentage of Bray–Curtis variation explained by PCoA1 was obtained from the PCoA eigenvalues. Spearman correlation was used to test the relationship between PCoA1 scores and root length; robustness was assessed by leave-one-out recalculation, with Pearson correlation used as an additional sensitivity check.

## 3. Results

### 3.1. Herbicide Treatments Induced Leaf-Level Physiological Stress Responses, with Differential Responses Between Rice and Barnyardgrass

**Figure 1:**
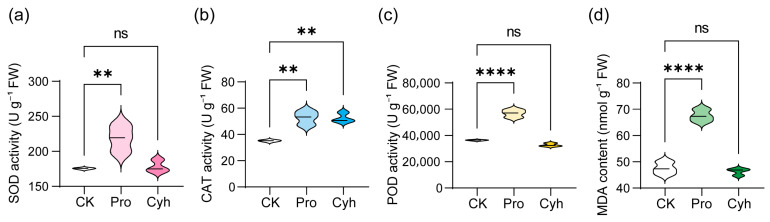
Effects of herbicide treatments on antioxidant enzyme activities and lipid peroxidation in barnyardgrass leaves under the rice–barnyardgrass co-cultivation system. (**a**–**d**) Leaf superoxide dismutase (SOD), catalase (CAT), and peroxidase (POD) activities, and malondialdehyde (MDA) content in barnyardgrass under control (CK), propanil (Pro), and cyhalofop-butyl (Cyh) treatments, respectively. Values are means ± SD (*n* = 3). ns indicates no significant difference; ** *p* < 0.01 and **** *p* < 0.0001 compared with CK, as determined by one-way ANOVA followed by Dunnett’s multiple comparisons test.

We first examined leaf physiological responses to sublethal Pro and Cyh treatments under co-cultivation by measuring antioxidant enzyme activities (SOD, POD, and CAT) and malondialdehyde (MDA) contents. At 24 h after treatment, Pro significantly increased SOD, POD, CAT, and MDA in barnyardgrass leaves relative to CK, whereas Cyh only significantly increased CAT (Figure 1a–d). Rice showed a different pattern: Pro increased SOD activity and decreased CAT and POD activities, whereas Cyh increased SOD activity and decreased POD activity (Figure S2). Neither herbicide significantly altered the MDA content in rice leaves under co-cultivation conditions (Figure S2), suggesting no clear evidence of overt lipid peroxidation. Thus, under co-cultivation, barnyardgrass exhibited a stronger oxidative stress-related response, whereas rice mainly exhibited antioxidant adjustment without significant MDA accumulation.

We then compared rice responses under monoculture. In monoculture, neither Pro nor Cyh significantly affected rice SOD activity, whereas both reduced CAT activity and increased POD activity relative to CK (Figure S3). Pro did not affect MDA, whereas Cyh significantly increased MDA under monoculture (Figure S3). This MDA increase was not accompanied by reduced growth traits, suggesting that it did not correspond to measurable growth inhibition during the observation period.

Untreated rice also differed with respect to baseline antioxidant profile between monoculture and co-cultivation (Figure S4). Together, these patterns indicate that physiological responses to herbicides in rice leaves were cultivation-context-dependent.

### 3.2. Herbicide Treatments Altered the Growth Response Patterns of Rice Under Rice–Barnyardgrass Co-Cultivation

**Figure 2:**
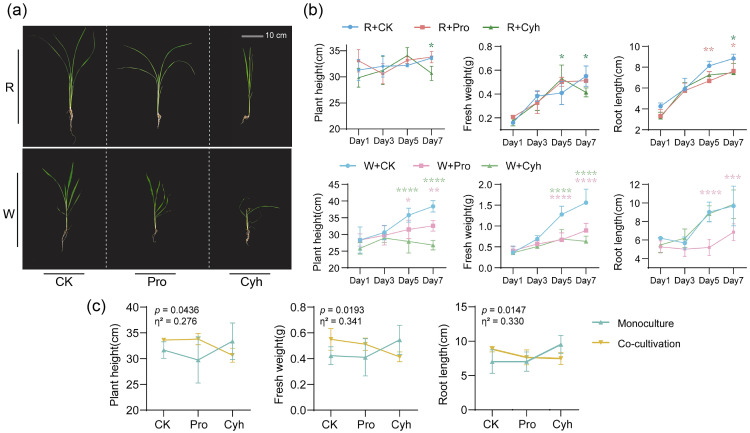
Growth responses of rice (R) and barnyardgrass (W) to herbicide treatments under the rice-barnyardgrass co-cultivation system. (**a**) Representative phenotypes of rice (R) and barnyardgrass (W) at 7 d after herbicide application under different herbicide treatments: CK (control), Pro (propanil), and Cyh (cyhalofop-butyl). Scale bar = 10 cm. (**b**) Temporal changes in plant height, fresh weight, and root length of rice (R) and barnyardgrass (W) at 1, 3, 5, and 7 d after herbicide application. Time-course data were analyzed by two-way ANOVA with treatment, sampling time, and their interaction as fixed factors. Within each time point, Pro and Cyh were compared with CK using one-way ANOVA followed by Dunnett’s test. Red and green asterisks denote Pro vs. CK and Cyh vs. CK, respectively (* *p* < 0.05, ** *p* < 0.01, *** *p* < 0.001, **** *p* < 0.0001). (**c**) Interaction effects of cultivation pattern (monoculture vs. co-cultivation) and herbicide treatment on rice growth traits. Two-way ANOVA results are shown, with *p* values indicating interaction significance and η^2^ representing effect size. Data are means ± SD (*n* = 4 pot-level biological replicates).

We next compared plant phenotypes and growth traits under co-cultivation at 1, 3, 5, and 7 d after herbicide application. Rice showed no visible phytotoxic symptoms, whereas barnyardgrass exhibited clear injury under both herbicides. Pro caused localized chlorosis and necrosis in barnyardgrass, while Cyh caused stronger systemic injury, including dwarfing, apical growth suppression, and leaf chlorosis (Figure 2a). This demonstrates that visible injury was much more pronounced in barnyardgrass than in rice.

Quantitative analysis of the time × treatment effects further showed sustained inhibition of barnyardgrass growth under both herbicide treatments during co-cultivation (Figure 2b). From day 5 onward, both herbicides significantly reduced plant height and shoot fresh weight in barnyardgrass, and Pro also reduced root length relative to CK. SPAD responses were transient and no longer differed from CK by day 7. Notably, the comparable biomass reductions observed at day 5 or 7 suggest that the two herbicide treatments were associated with a similar magnitude of overall growth response in the weed at the doses applied.

Despite the absence of visible injury, rice growth traits changed under herbicide treatment and co-cultivation conditions. Compared with the co-cultivation control, rice in the Cyh treatment group exhibited significantly lower plant height, shoot fresh weight, and root length at day 7. Shoot fresh weight showed a transient increase at day 5 before decreasing below CK at day 7. Meanwhile, Pro did not significantly affect rice plant height or shoot fresh weight but reduced the root length at days 5 and 7.

We then used rice monoculture and untreated co-cultivation controls to separate herbicide effects from the cultivation-context effects (Figure S5). Under monoculture, Pro did not significantly affect rice growth traits, whereas Cyh increased fresh weight and root length at day 7 (Figure S5). Under untreated and co-cultivation, rice growth was not suppressed. These findings indicate that the reduced growth of rice in the herbicide-treated co-cultivation system was associated with the combined context of herbicide application and rice–barnyardgrass co-cultivation, and was not consistently reproduced by herbicide treatment or co-cultivation alone.

Two-way ANOVA further supported an interactive effect between cultivation pattern and herbicide treatment on rice growth (Figure 2c). The interaction term between these factors was significant for plant height, fresh weight, and root length (*p* < 0.05), explaining 27.63%, 34.15%, and 33.04% of the variance, respectively. By contrast, the main effects of the individual factors were not significant. These results indicate that responses to herbicide treatment in rice growth depended on the cultivation pattern.

Root morphological traits showed a stronger and more sustained response in barnyardgrass than in rice (Figure S6). Pro reduced barnyardgrass root surface area, root volume, and root tip numbers across multiple diameter classes, whereas Cyh reduced these traits mainly in the 0.5–2 mm root tip classes. In rice, the root surface area, root volume, and average root diameter did not exhibit sustained significant changes, with only transient or isolated differences in root tip traits. Therefore, the suppression in rice growth was not accompanied by overt or sustained architectural damage to roots.

### 3.3. Co-Cultivation with Herbicide Treatment Reshaped the Rice Microbiome, with Herbicide-Specific Assembly Signals and Network Patterns

Given that rice growth suppression in the herbicide-treated co-cultivation system occurred without visible phytotoxicity or sustained root architectural damage, we next examined rhizosphere and root endosphere microbiomes. The analyses focused on diversity, community composition, βNTI-based assembly signals, and bacterial–fungal association networks.

**Figure 3:**
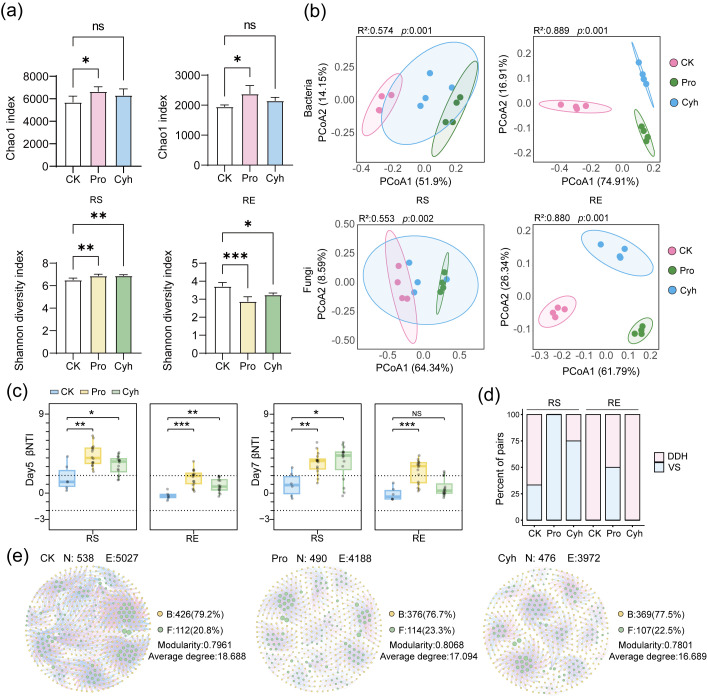
Effects of herbicide treatments on microbiome diversity, βNTI-based assembly patterns, and network structure in rice under co-cultivation. (**a**) Bacterial alpha diversity in rhizosphere soil (RS) and root endosphere (RE) at day 5. Bars show means ± SD. (**b**) Bray–Curtis PCoA of bacterial and fungal communities in RS and RE at day 5. Axis percentages indicate explained variation; PERMANOVA R^2^ and *p* values are shown. (**c**) Bacterial βNTI distributions in RS and RE at days 5 and 7. Dashed lines indicate βNTI = ±2. (**d**) Relative contributions of inferred assembly categories based on the βNTI–RCbray framework. VS, variable selection; DDH, drift- and dispersal-related stochastic categories. (**e**) Rhizosphere bacterial–fungal association networks at day 7. Yellow and green nodes indicate bacterial and fungal genera, respectively. N, nodes; E, edges; B, bacterial nodes; F, fungal nodes. CK, control; Pro, propanil; Cyh, cyhalofop-butyl. Asterisks indicate significant differences (* *p* < 0.05, ** *p* < 0.01, *** *p* < 0.001); ns, not significant.

Alpha-diversity responses were not continuous across the 7-day period. Changes on days 1 and 3 were sporadic and restricted to specific indices or compartments (Figure S7), whereas the most pronounced response was observed on day 5, particularly in bacterial communities (Figure 3a). On day 5, Pro increased rhizosphere bacterial richness, and both herbicides increased rhizosphere bacterial Shannon diversity. In the root endosphere, Pro increased bacterial richness, whereas both herbicides reduced the bacterial Shannon diversity. Fungal alpha-diversity responses were less consistent, and most diversity differences weakened by day 7. These patterns identify a period of 5 days as the main alpha-diversity response window, with bacteria showing the clearest compartment-dependent changes.

Bray–Curtis PCoA and PERMANOVA showed significant treatment-associated compositional shifts at day 5 in both rhizosphere soil and root endosphere (Figure 3b). Both Pro and Cyh communities differed from CK across bacterial and fungal datasets. The effect size was larger in the endosphere than in the rhizosphere, with PERMANOVA R^2^ values of 0.889 and 0.880 for endophytic bacterial and fungal communities, respectively, compared with 0.574 and 0.553 for rhizosphere bacterial and fungal communities. By day 7, rhizosphere separation had weakened, with Pro showing a more sustained effect than Cyh, whereas endophytic bacterial and fungal communities remained separated from CK under both herbicides. Thus, community compositional responses were strongest at day 5 and later became compartmentally differentiated.

βNTI analysis further indicated altered bacterial assembly signals under herbicide treatment and co-cultivation (Figure 3c). At day 5, rhizosphere bacterial communities in CK were mainly within the stochastic range, whereas Pro and Cyh increased βNTI values, indicating a shift toward stronger deterministic selection signals. This elevated rhizosphere βNTI pattern persisted to day 7 under both herbicides. In the root endosphere, bacterial βNTI responses were more herbicide-specific: Pro remained significantly different from CK at day 7, whereas Cyh did not show a stable deviation. These results indicate that bacterial community reorganization involved both compositional turnover and altered βNTI-based assembly signals.

Fungal βNTI patterns were weaker and less consistent, with no overall significant difference from CK despite some samples exceeding the ±2 threshold (Figure S8). Consistent with the bacterial βNTI distributions, the βNTI–RCbray summary highlighted a higher contribution of variable selection (VS) in herbicide-treated bacterial communities on day 5, whereas CK was more associated with drift- and dispersal-related stochastic categories (DDH), and homogeneous selection (HS) remained low (Figure 3d). Pro showed the highest VS contribution, particularly in the rhizosphere. Thus, both herbicides altered bacterial assembly signals, but Cyh was more associated with earlier diversity/compositional changes, whereas Pro showed a stronger deterministic-selection signal.

Additional day-5 comparisons showed that these βNTI patterns were not consistently reproduced by herbicide treatment under monoculture or by cultivation-mode differences alone (Figure S8). Across compartments and kingdoms, monoculture and co-cultivation comparisons mostly remained within the stochastic range and did not exhibit significant βNTI shifts. Thus, the bacterial shift toward stronger deterministic selection in the rhizosphere was most evident under the combined herbicide treatment–co-cultivation context.

Cross-kingdom bacterial–fungal networks also changed over time (Figure 3e). On day 5, rhizosphere networks under Pro and Cyh showed higher node and edge numbers than CK, indicating network reconfiguration rather than a uniform increase in compactness, as density and modularity were not consistently elevated (Figure S9). By day 7, both herbicide-treated rhizosphere networks exhibited lower node numbers, edge numbers, average degrees, and betweenness centralization than CK, indicating smaller and less connected putative association structures. Endophytic networks also showed treatment-associated differences in size, kingdom composition, and topology. These patterns were generally retained in cutoff sensitivity analyses and leave-one-out checks. Together, the network results suggest a transition from day-5 reconfiguration to day-7 simplification. Overall, the herbicide treatment–co-cultivation condition was associated with a concentrated day-5 microbiome response involving bacterial diversity shifts, compositional displacement, altered β NTI-based assembly signals, and network reconfiguration, followed by partial rhizosphere attenuation and day-7 network simplification.

### 3.4. Shared Responsive Genera Showed Host-Biased Redistribution Alongside Root Length-Associated Rhizosphere Community Variation on Day 5

**Figure 4:**
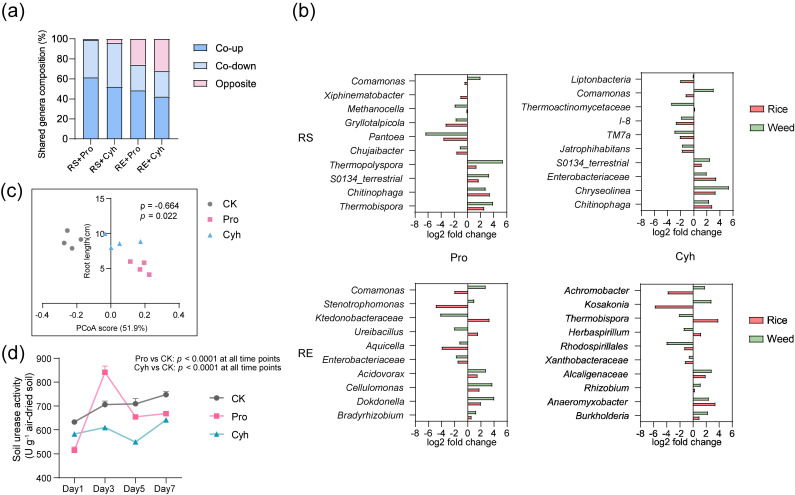
Shared responsive genera, root length-associated rhizosphere bacterial community structure, and enzyme activity under rice–barnyardgrass co-cultivation. (**a**) Shared responsive genera showing co-up, co-down, or opposite response patterns in RS and RE under Pro and Cyh treatments at day 5. Co-up and co-down indicate genera increasing or decreasing in both hosts relative to their corresponding controls; opposite indicates opposing response directions between hosts. (**b**) Representative shared responsive genera showing host-biased changes. Bars indicate log2 fold changes relative to the corresponding host-specific CK; positive and negative values indicate enrichment and depletion, respectively. (**c**) Association between rice root length and variation along PCoA1 of day-5 co-cultivated rice rhizosphere bacterial communities. PCoA was based on Bray–Curtis distances, and PCoA1 explained 51.9% of the variation. (**d**) Temporal changes in rhizosphere urease activity under CK, Pro, and Cyh treatments. Enzyme activity is expressed as U g^−1^ air-dried soil. Values are means ± SD (*n* = 4). *p* values indicate comparisons between herbicide treatments and CK at each time point. CK, control; Pro, propanil; Cyh, cyhalofop-butyl; RS, rhizosphere soil; RE, root endosphere.

As the most pronounced microbiome response was observed on day 5, we compared herbicide-responsive genera shared by rice and barnyardgrass under co-cultivation, with each host compared against its own CK. This shared-genera analysis was used to distinguish concordant responses from host-biased or opposing response patterns. In the rhizosphere, shared responsive genera were dominated by concordant changes, especially co-up responses, whereas opposite response directions accounted for a smaller fraction. In the root endosphere, the proportion of opposite responses increased, indicating stronger inter-host divergence within the internal host-associated niche (Figure 4a).

We then quantified this divergence by constructing a host-bias index based on differences in response magnitude between the barnyardgrass and rice sides. Under the Pro treatment, the shared responsive genera as a whole exhibited a significant bias toward the barnyardgrass side, and this tendency was more consistently observed among highly abundant shared genera as well as those that responded significantly in both hosts. Under Cyh treatment, however, the overall bias was comparatively weak and no consistent directional shift emerged at the community level. Taken together, these results indicate that the microbial response under herbicide application and co-cultivation did not appear to be a simple synchronous perturbation of the shared microbiota, but was accompanied by an asymmetric redistribution of shared taxa between the two hosts, with host differentiation being especially pronounced in the endosphere.

Analyses of representative taxa supported this host-biased pattern (Figure 4b). *Comamonas* showed a consistent barnyardgrass-side bias in both the rhizosphere and endosphere under Pro and in the rhizosphere under Cyh. In contrast, *Thermoactinomycetaceae* showed a rice-side bias in the rhizosphere under Cyh. Several genera, including *Stenotrophomonas*, *Achromobacter*, and *Kosakonia*, exhibited stronger inter-host differences in the endosphere. These examples indicate that shared microbiota did not respond synchronously across hosts but included taxa with stable directional differences.

To test whether this microbial reorganization was reflected in early rice performance, we next examined the relationship between overall rhizosphere bacterial community variation and the earliest responsive rice growth trait at day 5. Because root length was the first rice trait to show a significant response, we related root length to variation along the major axis of rhizosphere bacterial community structure. Across day-5 co-cultivated rice samples from CK, Pro, and Cyh treatments, root length was negatively correlated with PCoA1 scores of rhizosphere bacterial communities (Spearman’s ρ = −0.664, *p* = 0.022; Figure 4c). PCoA1 explained 51.9% of the Bray–Curtis variation. The negative direction was retained in leave-one-out analysis (ρ = −0.809 to −0.564), and Pearson correlation showed the same relationship (r = −0.701, *p* = 0.011). These results suggest that variation along the major bacterial community axis was temporally associated with the early root response of rice.

We further assessed whether these community and plant responses were accompanied by changes in enzyme-based indicators of rhizosphere biochemical activity. The two enzyme indicators showed distinct response patterns rather than a uniform shift. Urease activity differed significantly from CK under both herbicide treatments throughout the observation period and generally remained lower during the later stages (Figure 4d). Acid phosphatase activity showed a more transient response, increasing under both Pro and Cyh treatments at day 5 but showing no significant differences at the other sampling times (Figure S10). Together, these results indicate that herbicide treatment under co-cultivation was accompanied by detectable, indicator-dependent shifts in measured rhizosphere enzyme activities, including a sustained urease-related response and a stage-specific acid phosphatase response.

## 4. Discussion

Herbicide selectivity in rice production is commonly inferred from the contrast between crop tolerance and weed suppression, particularly visible crop injury, growth performance, and field efficacy [17,33]. Propanil and cyhalofop-butyl are generally considered selective to rice because rice can rapidly detoxify these compounds, thereby limiting direct phytotoxicity at recommended doses [18,34]. Our results do not contradict the established physiological basis of rice tolerance, because rice showed no severe visible injury and barnyardgrass remained more strongly affected. However, they show that apparent crop tolerance was strongly context dependent. The same sublethal treatments that caused limited rice responses in monoculture suppressed rice growth under rice-barnyardgrass co-cultivation, and this response was supported by significant cultivation pattern × herbicide interactions. Thus, reduced rice growth was not attributable to herbicide exposure or interspecific coexistence alone, but arose within their combined context. This finding extends the understanding of herbicide selectivity from individual crop tolerance to crop–weed settings, where responses are shaped by neighboring plants and belowground biota [15]. Such context-dependent outcomes are consistent with broader plant–soil studies showing that stress responses depend not only on the stressor but also on the surrounding biological environment, including neighboring plants and associated microbiota [35,36]. The combined context likely included several non-exclusive components, including direct sublethal effects on rice, herbicide-induced changes in barnyardgrass status, altered crop–weed interaction intensity and changes in root-derived inputs and rhizosphere conditions.

The microbiome data reveal that the belowground response is an early and stage-dependent component of the herbicide-co-cultivation context, rather than a uniform or delayed microbiome shift. Within the 7-day sampling scheme, the clearest pronounced response was observed on day 5: diversity shifts, compositional displacement, βNTI-based assembly signals, and network reconfiguration were all evident at this stage, whereas the strongest decline in rice growth was observed later. This temporal ordering is important because microbial changes detected before or during the onset of host phenotypic decline are less likely to represent only secondary consequences of plant deterioration [37,38]. Bacterial communities showed clearer and more consistent responses than fungal communities, particularly in diversity, composition, and βNTI-based signals. This difference is ecologically plausible because bacteria often respond rapidly to short-term changes in root-derived substrates and local physicochemical conditions, whereas fungal communities may be buffered by slower turnover, hyphal persistence, or greater structural stability [39].

The rhizosphere bacterial βNTI pattern provides a useful phylogeny-based view of this response and is best interpreted as an inferred assembly signal. In untreated co-cultivation controls, rhizosphere bacterial communities remained largely within the stochastic range, and βNTI–RCbray partitioning indicated a greater contribution of drift- and dispersal-related categories [40]. In contrast, both herbicides led to increased βNTI values and increased the contribution of variable selection at day 5, suggesting a stronger deterministic selection signal in herbicide-treated co-cultivation systems [40]. Propanil showed a particularly clear shift away from stochastic dominance, whereas cyhalofop-butyl was more associated with earlier diversity and compositional changes. These herbicide-specific patterns may reflect differences in how the two compounds alter the local selection environment for belowground bacteria, which may occur through root-derived inputs, barnyardgrass suppression intensity, or the physiological status of neighboring hosts [41]. Importantly, the broader βNTI shift was not consistently reproduced by herbicide treatment under monoculture or by cultivation-mode differences alone. Therefore, the deterministic-selection signal in rhizosphere bacteria was most evident when chemical disturbance occurred within the rice–barnyardgrass co-cultivation context.

The endosphere exhibited a related but more host-dependent pattern. Compared with the rhizosphere, endophytic communities displayed stronger treatment-associated compositional shifts and, in some cases, retained separation from CK even after rhizosphere responses had weakened. This compartmental contrast suggests that the endosphere did not simply mirror transient changes at the soil–root interface, but retained a more host-filtered disturbance signal [42]. This is relevant in the rice–barnyardgrass system because the two hosts recruit from the same soil microbial reservoir but diverged physiologically after herbicide exposure.

The shared-genus analysis further supported host-biased redistribution of the shared microbiota. Rhizosphere responses were dominated by concordant changes, consistent with a broadly shared perturbation at the external soil interface. In contrast, opposing responses were more frequent in the endosphere, indicating stronger host-dependent divergence in the direction and magnitude of shared-taxon responses. Under propanil, this bias was oriented mainly toward the barnyardgrass side. Representative genera also showed directional asymmetry: Comamonas was repeatedly biased toward barnyardgrass across treatments or compartments, whereas Herbaspirillum showed a rice-biased pattern under cyhalofop-butyl. These taxa have been reported in denitrification-related or plant-associated endophytic contexts, respectively [43,44], but their roles in the present system remain inferential. The shared microbiota did not respond as a uniform pool; instead, the herbicide-treated co-cultivation system was associated with host-biased redistribution, especially in the endosphere, where microbial communities are more tightly shaped by host selection [42,45]. Although individual taxa were not experimentally manipulated, these host-biased patterns indicate that shared microbiota formed part of the asymmetric ecological response under herbicide treatment–co-cultivation conditions.

Association-network patterns were consistent with this staged reorganization. On day 5, rhizosphere bacterial–fungal networks exhibited increased node and edge numbers under both herbicides, whereas density and modularity were not consistently elevated. This pattern is better interpreted as network reconfiguration than as strengthened microbial interactions. By day 7, herbicide-treated rhizosphere networks became smaller and less connected, suggesting that early reconfiguration was followed by a simplified putative association structure. Because co-occurrence networks infer statistical associations rather than direct interactions, these patterns are best interpreted as reorganization of the potential bacterial–fungal association structure.

Microbiota reorganization was linked to a biochemical dimension of the rhizosphere response, as reflected by changes in measured enzyme activities. Urease activity showed a more sustained deviation under herbicide treatment, whereas acid phosphatase showed only stage-specific fluctuations. This contrast suggests that the enzyme-level response was selective rather than uniformly suppressive [46,47]. Together with the significant negative association between rice root length and the major rhizosphere bacterial community axis on day 5, these patterns link microbial reorganization with early responses in rice roots and rhizosphere biochemical change. However, this evidence remains correlative. Microbiome manipulation, microbial rescue, or synthetic-community experiments will be needed to determine whether microbial changes contributed causally to the suppression in rice growth.

Within this common co-cultivation-dependent response, the two herbicides nevertheless showed different response profiles. Cyhalofop-butyl coincided with earlier bacterial diversity and compositional shifts and stronger aboveground growth suppression of rice at day 7, whereas propanil showed a clearer βNTI-based deterministic-selection signal and a stronger barnyardgrass-side bias among shared responsive genera. Consistent with the growth data, propanil was also the treatment that more clearly reduced rice root length at days 5 and 7. These differences suggest that herbicides with distinct modes of action may generate different combinations of physiological and ecological responses in crop-weed-microbiome systems [48]. Nevertheless, both treatments led to the same broader implication: apparent rice safety under monoculture did not predict the plant-level and microbiological responses observed under crop–weed coexistence. This highlights the importance of interpreting herbicide selectivity within an active crop–weed–microbiome context [49,50].

Several limitations should be considered. First, the experiment covered only the first 7 days after herbicide application; therefore, day 5 should be interpreted as a short-term response window within the present sampling scheme rather than a validated temporal threshold. Longer time-course studies with denser sampling around days 3–7 are needed to test this duration and its reproducibility. Second, the experiment was conducted in pots under controlled conditions, and field studies incorporating hydrological variation, soil heterogeneity, and agronomic management are required to evaluate field relevance. From an applied perspective, our findings suggest that herbicide selectivity in paddy systems should be interpreted with attention to microbiome-linked crop–weed responses, rather than relying only on dose, direct crop tolerance, or weed suppression. Future intervention experiments could test whether maintaining rhizosphere microbial stability, for example through microbial inoculation or management practices that preserve beneficial rhizosphere consortia, can mitigate such context-dependent responses [51].

## 5. Conclusions

This study shows that rice responses to sublethal herbicide exposure were strongly shaped by the rice–barnyardgrass co-cultivation context. Although rice exhibited no visible phytotoxicity, Pro and Cyh suppressed its rice growth under co-cultivation, whereas comparable suppression was not observed under herbicide exposure alone or co-cultivation alone. Barnyardgrass, in contrast, exhibited higher oxidative stress and visible injury, confirming the weed-directed activity of these treatments. These findings indicate that reduced rice growth was associated with the combined herbicide treatment–co-cultivation context rather than with either factor alone. The microbiome results further show that herbicide exposure under co-cultivation initiated an early and stage-specific belowground response. Microbial changes were detectable before the clearest rice growth decline and became most pronounced on day 5, mainly in bacterial communities. Herbicide-treated co-cultivation altered microbial diversity, community composition, βNTI-based assembly signals, bacterial–fungal association networks, and shared responsive genera between rice and barnyardgrass. The negative association between bacterial community variation and rice root length, together with altered rhizosphere enzyme activities, further links microbiota reorganization with early rice growth performance and rhizosphere biochemical change. Overall, these results indicate that herbicide selectivity in rice–barnyardgrass systems should be interpreted within a crop–weed–microbiome context, because microbial reorganization forms an important part of the ecological response to herbicide disturbance under co-cultivation.

## Data Availability

The data presented in this study are openly available in the NCBI Sequence Read Archive (SRA) under BioProject accession number PRJNA1449069. The public BioProject record will be available at https://www.ncbi.nlm.nih.gov/bioproject/PRJNA1449069 (accessed on 12 May 2026). The associated code supporting this study has been deposited in Zenodo: https://doi.org/10.5281/zenodo.19568375 (accessed on 12 May 2026).

## Supplementary Materials

The following supporting information can be downloaded at: https://www.mdpi.com/article/10.3390/microorganisms14051137/s1, Figure S1: OTU rarefaction curves for bacterial and fungal communities. (a) Bacterial communities. (b) Fungal communities. Each curve represents one retained sequencing sample. Sample labels correspond to the original sequencing sample IDs used during bioinformatic processing. Curves approached saturation, indicating that the sequencing depth was sufficient for downstream diversity analyses; Figure S2: Activities of antioxidant enzymes and MDA content in leaves of co-cultivated rice under different treatments. SOD, superoxide dismutase; CAT, catalase; POD, peroxidase; MDA, malondialdehyde. Data are presented as mean ± SD. Statistical significance is indicated as * *p* < 0.05, ** *p* < 0.01, *** *p* < 0.001 and **** *p* < 0.0001; ns, not significant. CK, control; Pro, propanil; Cyh, cyhalofop-butyl; Figure S3: Leaf antioxidant enzyme activities and MDA content of monocultured rice under different herbicide treatments. Data are presented as mean ± SD. * *p* < 0.05, ** *p* < 0.01, *** *p* < 0.001; ns, not significant; Figure S4: Comparison of leaf antioxidant enzyme activities and MDA content between monocultured and co-cultivated rice without herbicide treatment. Data are presented as mean ± SD. * *p* < 0.05, *** *p* < 0.001; ns, not significant; Figure S5: Comparison of plant height, fresh weight, and root length between monocultured and co-cultivated rice without herbicide treatment (a–c), and among different herbicide treatments in monocultured rice (d–f). Data are presented as mean ± SD. * *p* < 0.05, ** *p* < 0.01; ns, not significant; Figure S6: Root morphological traits of co-cultivated rice under different herbicide treatments. (a) Root volume, average root diameter, and root surface area. (b) Numbers of root tips in different diameter classes (0–0.2, 0.2–0.5, 0.5–1, 1–2, and >2 mm). Data are presented as mean ± SD. * *p* < 0.05, ** *p* < 0.01; ns, not significant; Figure S7: Alpha diversity of rhizosphere and root endophytic microbial communities at Day 1 and Day 3 under different herbicide treatments. (a) Rhizosphere bacteria; (b) rhizosphere fungi; (c) root endophytic bacteria; (d) root endophytic fungi. Chao1 and Shannon indices are shown. Data are presented as mean ± SD. * *p* < 0.05, ** *p* < 0.01, *** *p* < 0.001; ns, not significant; Figure S8: βNTI values of rhizosphere (RS) and root endophytic (RE) microbial communities under different treatments. (a) Fungal βNTI of co-cultivated rice in RS and RE at Day 5 and Day 7 under different herbicide treatments; (b) bacterial and fungal βNTI of monocultured rice in RS and RE at Day 5 and Day 7 under different herbicide treatments; (c) bacterial and fungal βNTI values of monocultured and co-cultivated rice in RS and RE at Day 5 and Day 7 without herbicide treatment. Data are shown as boxplots; ns, not significant. * *p* < 0.05; Figure S9: Rhizosphere bacterial–fungal association networks at day 5 under herbicide-treated co-cultivation. Networks are shown for (a) CK, (b) Pro, and (c) Cyh. Yellow and green nodes indicate bacterial and fungal taxa, respectively. N, nodes; E, edges; B, bacterial nodes; F, fungal nodes; M, modularity; A, average degree. CK, control; Pro, propanil; Cyh, cyhalofop-butyl; Figure S10: Acid phosphatase activity in the rhizosphere soil of co-cultivated rice at Day 1, Day 3, Day 5, and Day 7 under different herbicide treatments. Data are presented as mean ± SD. * *p* < 0.05; ns, not significant.
