# Supplementary material for: Herbicide Application Under Co-Cultivation Is Associated with Early Microbiome Assembly Shifts and Later Physiological Decline in Rice
Source: Microorganisms. 2026 May 17;14(5):1137. doi: 10.3390/microorganisms14051137 (PMC13209617; doi:10.3390/microorganisms14051137)
Supplement: Supplementary file 1 [file microorganisms-14-01137-s001.zip › Supplementary_Figures.pdf]

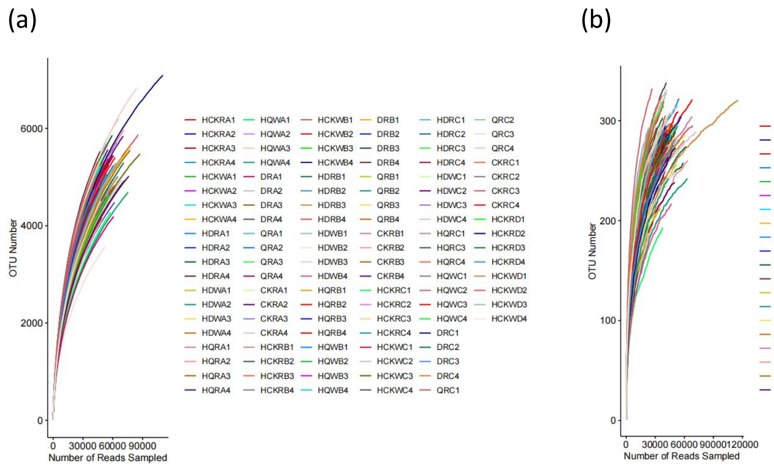

Figure S1. OTU rarefaction curves for bacterial and fungal communities.

(a) Bacterial communities. (b) Fungal communities. Each curve represents one retained sequencing sample. Sample labels correspond to the original sequencing sample IDs used during bioinformatic processing. Curves approached saturation, indicating that the sequencing depth was sufficient for downstream diversity analyses.

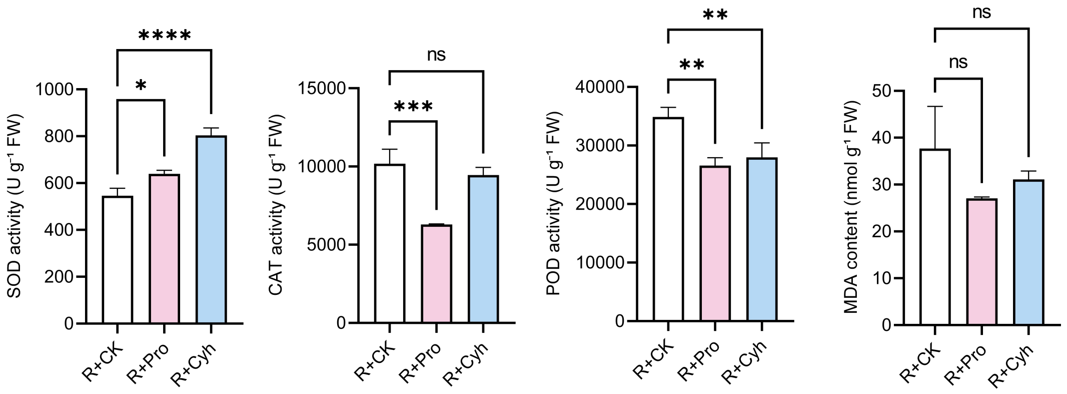

Figure S2. Activities of antioxidant enzymes and MDA content in leaves of co-cultivated rice under different treatments.

SOD, superoxide dismutase; CAT, catalase; POD, peroxidase; MDA, malondialdehyde. Data are presented as mean ± SD. Statistical significance is indicated as * $p < 0.05$, ** $p < 0.01$, *** $p < 0.001$ and **** $p < 0.0001$; ns, not significant. CK, control; Pro, propanil; Cyh, cyhalofop-butyl.

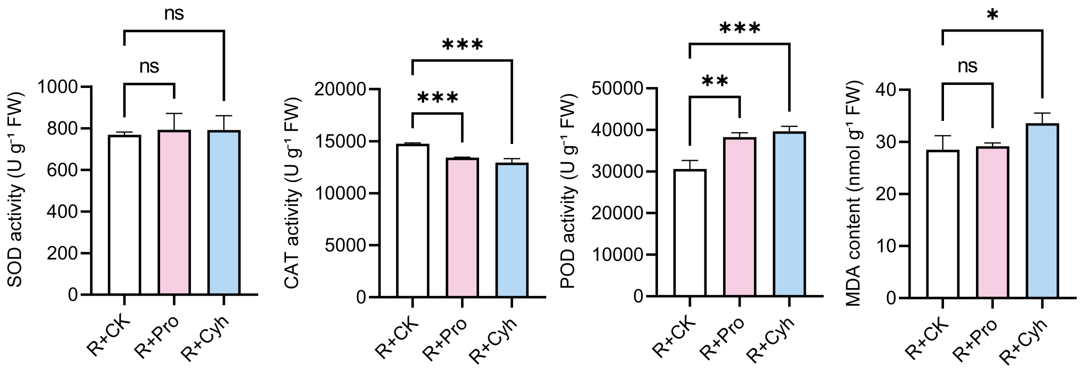

Figure S3. Leaf antioxidant enzyme activities and MDA content of monocultured rice under different herbicide treatments.

Data are presented as mean ± SD. * $p < 0.05$, ** $p < 0.01$, *** $p < 0.001$; ns, not significant.

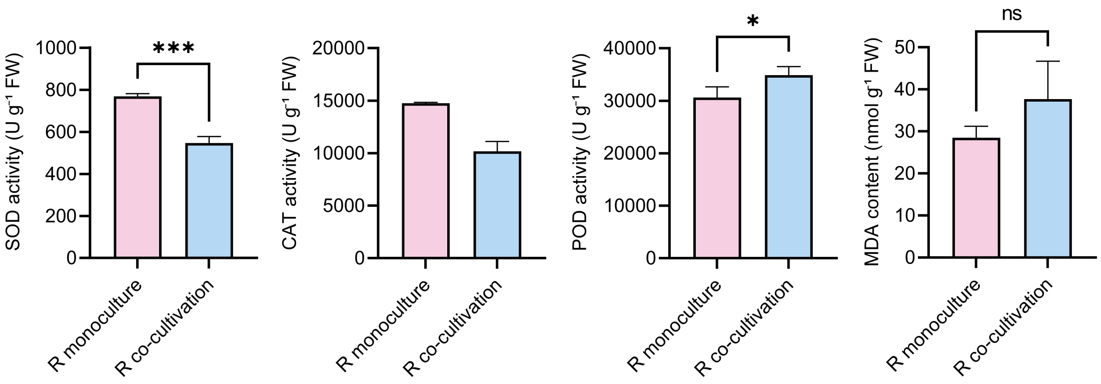

Figure S4. Comparison of leaf antioxidant enzyme activities and MDA content between monocultured and co-cultivated rice without herbicide treatment.

Data are presented as mean ± SD. * $p < 0.05$, *** $p < 0.001$; ns, not significant.

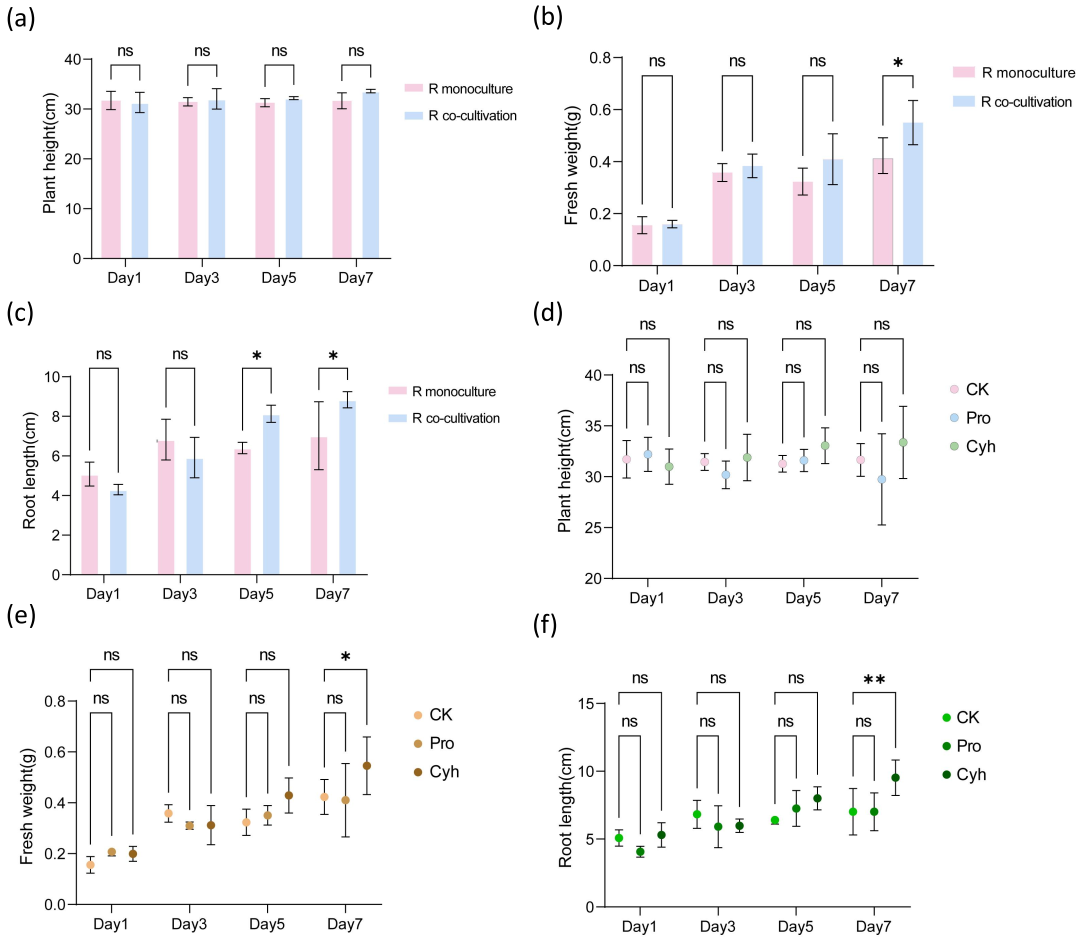

Figure S5. Comparison of plant height, fresh weight, and root length between monocultured and co-cultivated rice without herbicide treatment

(a–c), and among different herbicide treatments in monocultured rice (d–f). Data are presented as mean ± SD. * $p < 0.05$, ** $p < 0.01$; ns, not significant.

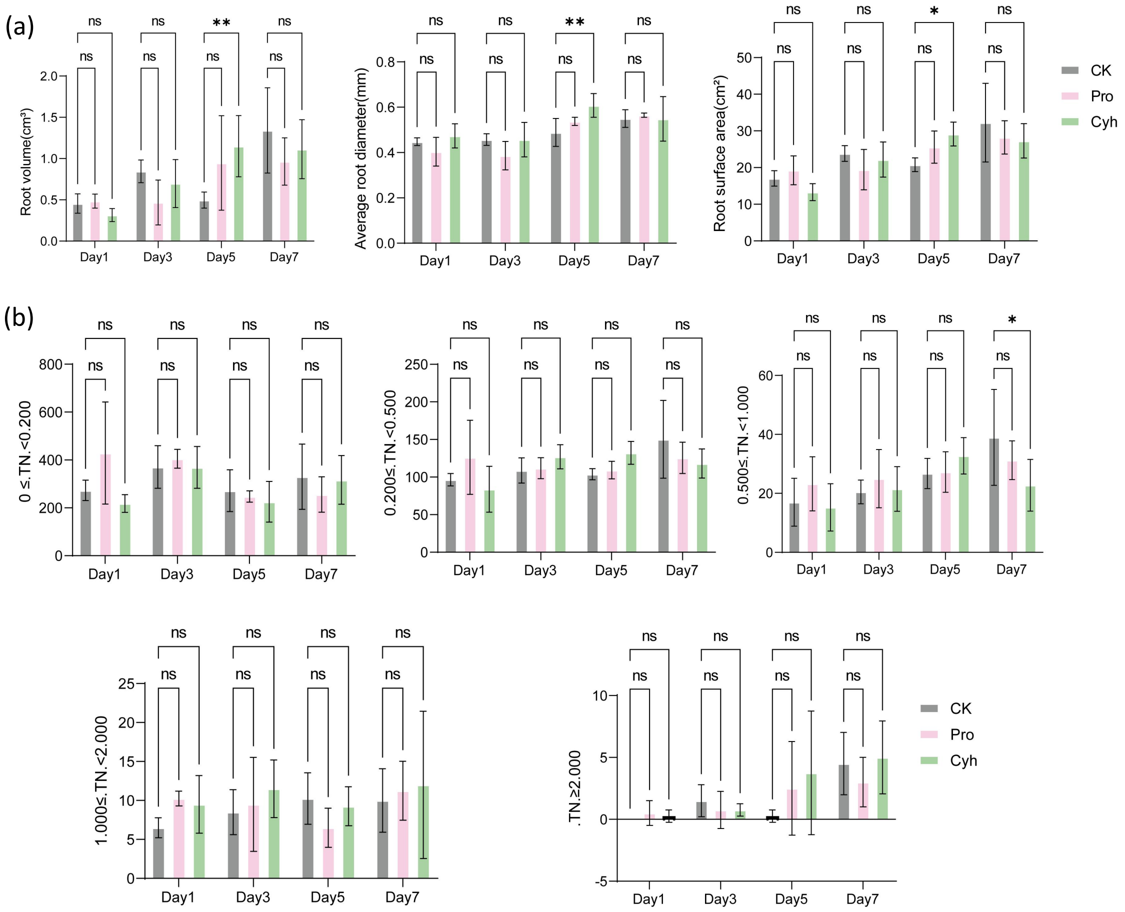

Figure S6. Root morphological traits of co-cultivated rice under different herbicide treatments. (a) Root volume, average root diameter, and root surface area. (b) Numbers of root tips in different diameter classes (0–0.2, 0.2–0.5, 0.5–1, 1–2, and >2 mm). Data are presented as mean ± SD. * $p < 0.05$, ** $p < 0.01$; ns, not significant.

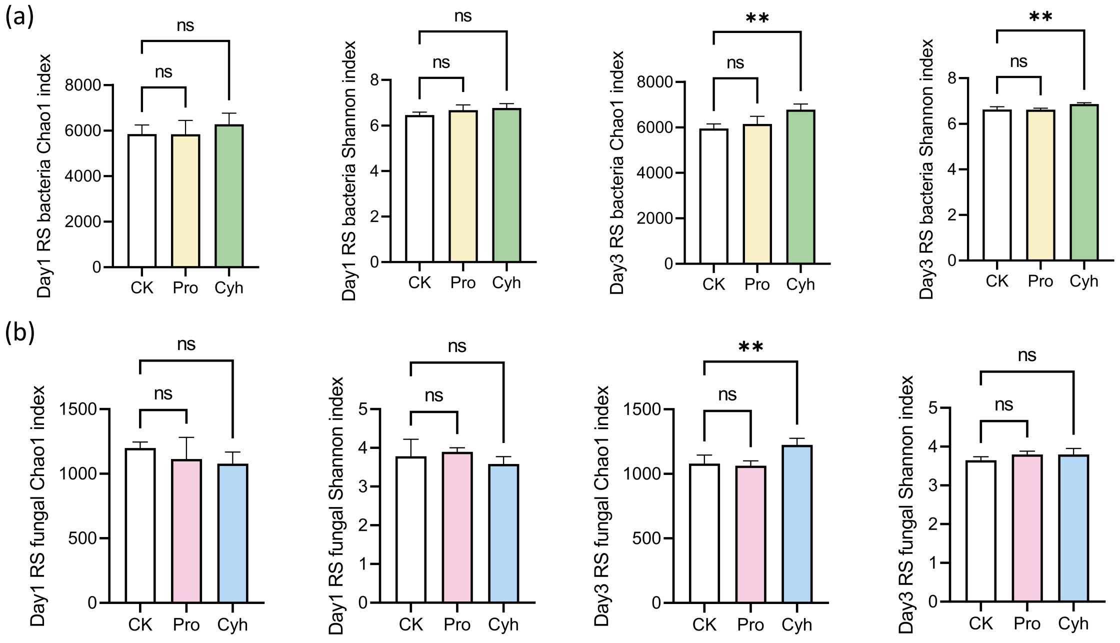

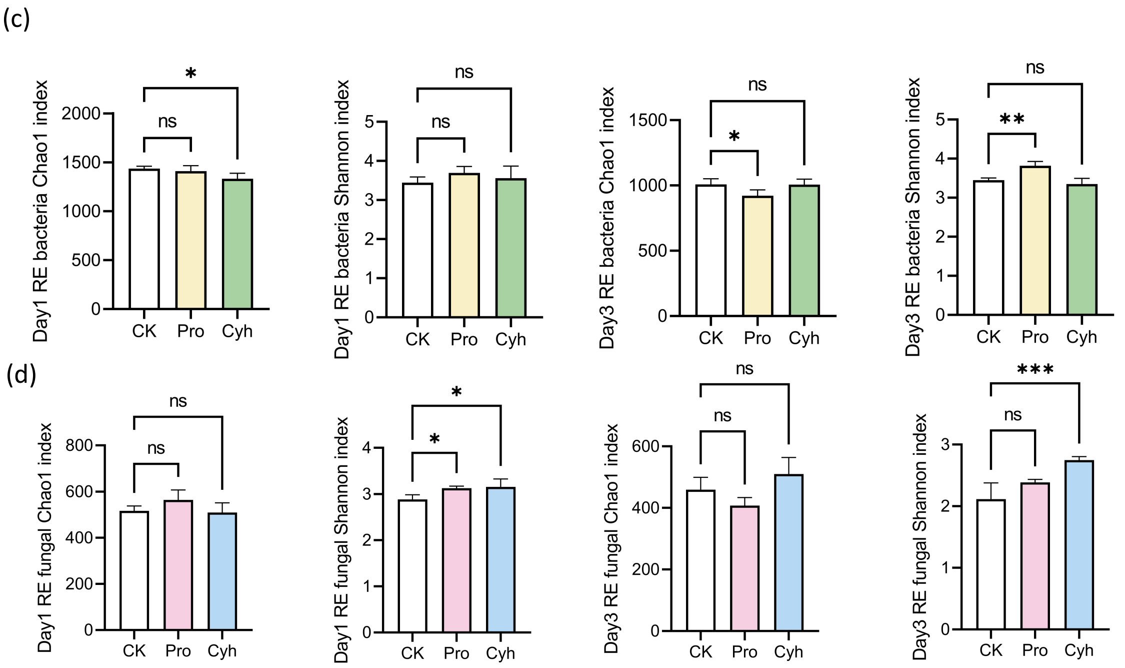

Figure S7. Alpha diversity of rhizosphere and root endophytic microbial communities at Day 1 and Day 3 under different herbicide treatments.

(a) Rhizosphere bacteria; (b) rhizosphere fungi; (c) root endophytic bacteria; (d) root endophytic fungi. Chao1 and Shannon indices are shown. Data are presented as mean ± SD. * $p < 0.05$, ** $p < 0.01$, *** $p < 0.001$; ns, not significant.

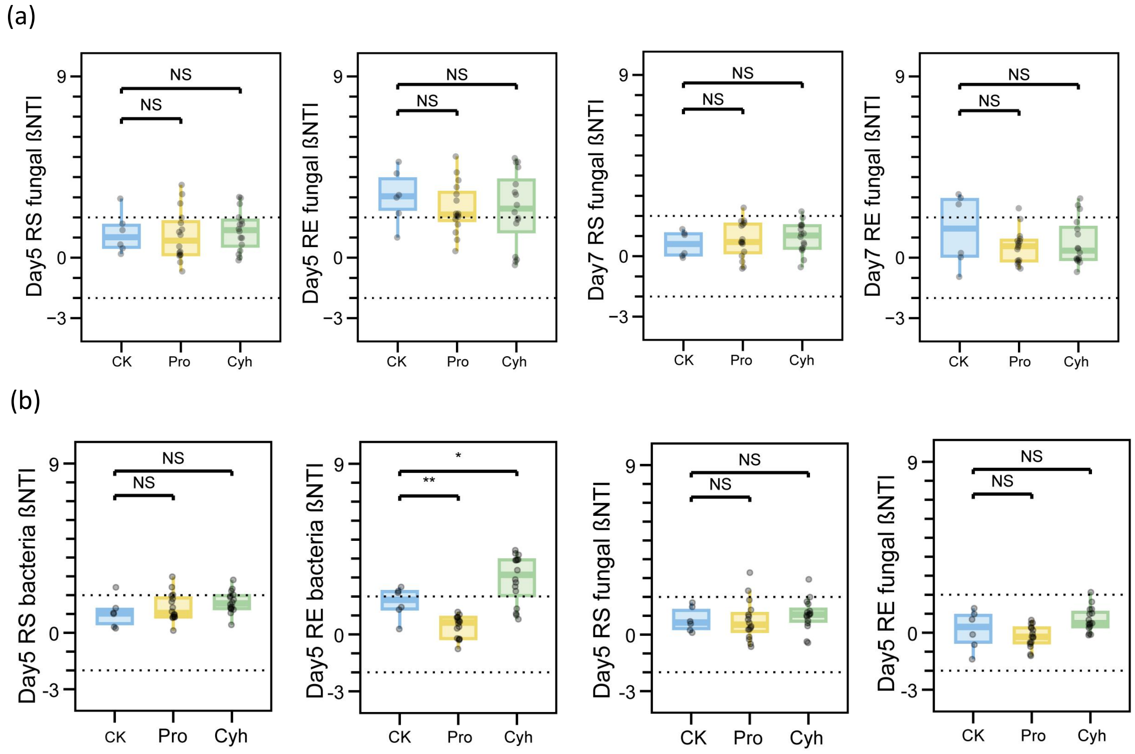

(c)

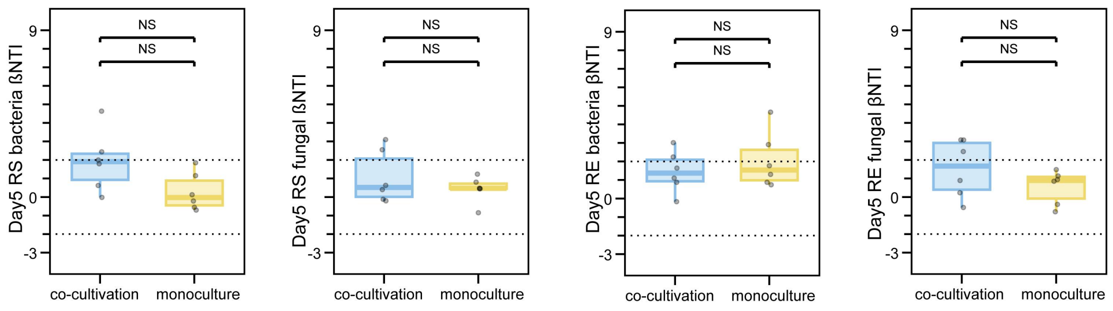

Figure S8. βNTI values of rhizosphere (RS) and root endophytic (RE) microbial communities under different treatments. (a) Fungal βNTI of co-cultivated rice in RS and RE at Day 5 and Day 7 under different herbicide treatments; (b) bacterial and fungal βNTI of monocultured rice in RS and RE at Day 5 and Day 7 under different herbicide treatments; (c) bacterial and fungal βNTI values of monocultured and co-cultivated rice in RS and RE at Day 5 and Day 7 without herbicide treatment. Data are shown as boxplots; ns, not significant.* $p < 0.05$.

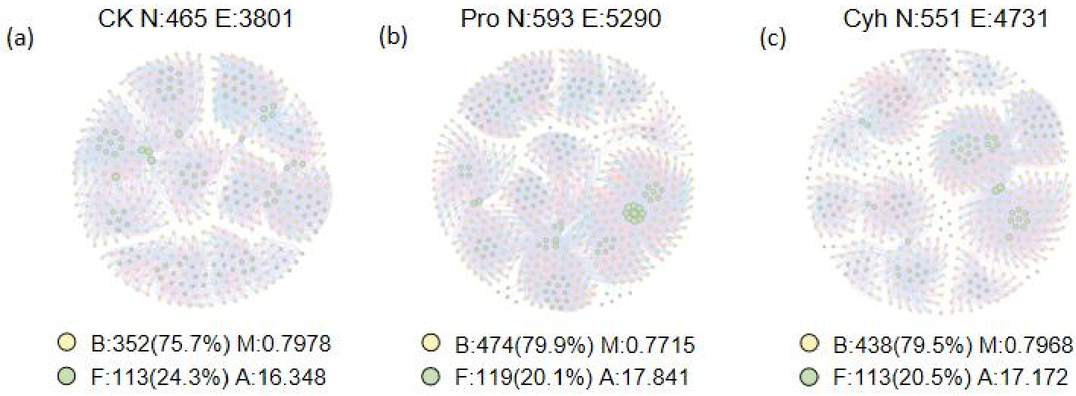

Figure S9. Rhizosphere bacterial-fungal association networks at day 5 under herbicide-treated co-cultivation.

Networks are shown for (a) CK, (b) Pro, and (c) Cyh. Yellow and green nodes indicate bacterial and fungal taxa, respectively. N, nodes; E, edges; B, bacterial nodes; F, fungal nodes; M, modularity; A, average degree. CK, control; Pro, propanil; Cyh, cyhalofop-butyl.

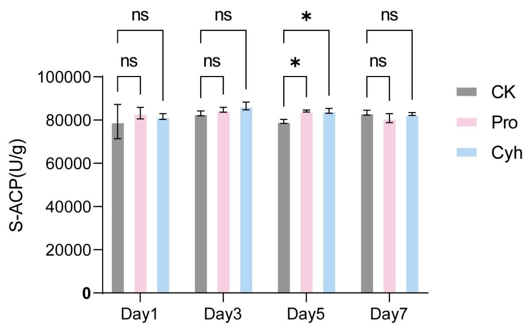

Figure S10. Acid phosphatase activity in the rhizosphere soil of co-cultivated rice at Day 1, Day 3, Day 5, and Day 7 under different herbicide treatments.

Data are presented as mean ± SD. * $p < 0.05$; ns, not significant.
